# Association of tobacco retailer count with smoking population versus vaping population in California (2019)

**DOI:** 10.1186/s13690-022-00799-1

**Published:** 2022-01-27

**Authors:** Vidya Purushothaman, Raphael E. Cuomo, Jiawei Li, Matthew Nali, Tim K. Mackey

**Affiliations:** 1 Department of Anesthesiology, San Diego School of Medicine, University of California, La Jolla, CA USA; 2 Global Health Policy and Data Institute, San Diego, CA USA; 3 S-3 Research LLC, San Diego, CA USA; 4 Global Health Program, Department of Anthropology, University of California, San Diego, La Jolla, CA USA

**Keywords:** Tobacco, Vaping, Smoking prevalence, Tobacco Retail, Ecological study

## Abstract

**Background:**

Access to tobacco products, including vape products, from local brick-and-mortar stores influences the exposure, uptake, and use of these products in local communities.

**Methods:**

Licensed tobacco retailers in California were classified as specialized tobacco/vape stores or non-specialized stores by obtaining categories published on Yelp. California smoking and vaping prevalence data were obtained from the 500 cities project and ESRI community analyst tool respectively. A series of simple linear regression tests were performed, at the zip code level, between the retailer count in each store category and smoking/vaping population. The Getis-Ord Gi* and Anselin Local Moran’s I statistics were used for characterization of tobacco retail density hotspots and cold spots.

**Results:**

The association between CA smoking/vaping population and number of tobacco retailers was statistically significant for all store categories. Variability in smoking population was best explained by variability in non-specialized storefronts(R^2^=0.84). Spatial variability in tobacco-only storefronts explained the least proportion of variability in the overall smoking population. Similar results were obtained specific to vaping population, although the proportion of population explained by variability in the number of non-specialized storefronts was comparatively lower(R^2^=0.80).

**Conclusions:**

Localities with greater numbers of non-specialized tobacco retailers had higher rates of smoking/vaping populations, and this association was much stronger for localities with greater numbers of specialized retailers. Non-specialized storefronts may represent convenient access points for nicotine products, while specialized storefronts may represent critical access points for initiation. Hence, regulations that address the entirety of the tobacco/vaping retail environment by limiting widespread access from non-specialized stores and reducing appeal generated by specialized retailers should be incorporated in future tobacco regulatory science and policymaking.

## Background

National and state-specific growth in the number of retail outlets for the tobacco industry enables easier access to conventional (e.g., cigarettes, cigars, little cigars, and cigarillos) and emerging tobacco products (E-cigarettes, IQOS) especially among youth and young adults [1]. Furthermore, the tobacco retail landscape is constantly evolving, with the number of access points now expanding to include specialty stores that exclusively focus on the distribution, marketing, and sale of popular vaping products along with generating demand for other alternative and emerging tobacco products (e.g., JUUL, Heat-Not-Burn) [2].

In the United States, the proportion of youth and young adults now using vaping products has increased considerably [3], leading to greater demand and increased diversity of retailers specializing and exclusively catering to the vaping community. Also, 58.8% of e-cigarette users were also current cigarette smokers in the United States [4]. While 12% of California adults (about 3.6 million) reported current use of one or more tobacco products, cigarette was the most reported tobacco product used, followed by e-cigarettes [5]. The rapid growth in vaping popularity over the past decade [6] has also led to renewed concerns about nicotine addiction, poisoning, and associated adverse events (including the 2019 e-cigarette, or vaping, product use-associated lung injury outbreak) [7], drawing greater public scrutiny to increased vaping product marketing and accessibility. Specifically, market access to new and diverse vaping products can lead to never smokers initiating, current users transitioning, and also possible dual product use (i.e., both cigarettes and vape products) [8, 9].

Prior studies have suggested that geographic proximity to a tobacco retailer is associated with higher smoking prevalence and lower cessation attempts [10]. Smoking prevalence is also higher among certain demographic and socioeconomic groups, such as those below poverty line, individuals with lower educational attainment, and minority groups such as American Indian/Alaska Natives [11] leading to tobacco and health-related disparities [12]. This is further worsened through aggressive point-of-sale advertisements, where in 2018 alone, the tobacco industry spent over 7.2 billion dollars on retail marketing and promotion of their products [13]. In addition, while the vaping population has increased, research conducted by the U.S. Centers for Disease Control and Prevention (CDC) found that most vape users were not successful in quitting and instead transitioned to dual use [4], further aggravating health outcomes for vaping initiating populations.

In the United States, requiring tobacco retailers to apply for licenses has been used as a form of regulation by state and local governments. As of 2019, 38 states require a license to sell tobacco products direct-to-consumer [14]. In California, every retailer who sells cigarettes or tobacco products is required to obtain a retailer’s license from the California Department of Tax and Fee Administration (CDTFA) and renew it annually (in accordance with the California Cigarette and Tobacco Products Licensing Act of 2003). In June 2016, CA state law expanded the definition of tobacco products to specifically include electronic smoking or vaping devices that deliver nicotine or other vaporized liquids, resulting in all vaping retailers being required to obtain a license from the CDTFA. Although the CDTFA listing of licensed tobacco retailers is publicly available, it does not distinguish between specialized tobacco and/or vape stores and non-specialized stores (e.g., general retailers, convenience stores, gas stations, etc.).

Yelp is a popular crowd-sourced business listing website that provides business information, location, store ratings and reviews, service/product availability and category for the store’s primary business line. For tobacco retailers, Yelp may categorize stores as vape shops or tobacco shops, but also as a grocery store, convenience store, gas station, or other general retailer. Therefore, using Yelp labelled categories, specialized tobacco and/or vape shops can be distinguished from non-specialized retailers that are licensed to sell tobacco products by the CDTFA.

Past research has examined the association between tobacco retail density with state-level smoking rates [10] and county-level daily smoking odds [15]. Additionally, small area estimates are used to better understand health behavior related variables in place-based research [16]. Prior studies have used small area estimates to observe the place-based inequities in smoking prevalence in the largest cities in the United States [17], explore the association between vape shop locations and young adult tobacco use [18], and describe the age disparities in tobacco retail density of specialized and non-specialized storefronts [19]. However, less is known about the impact of tobacco retail exposure on small area estimates of smoking and vaping prevalence when stratified specifically for specialized tobacco and/or vape stores compared to non-specialized storefronts. To address this research gap, this exploratory ecological study analyzes variations in the association between California tobacco retail store categories and retailer count with levels of smoking and vaping population at the zip code level.

## Methods

### Data Collection

A list of licensed tobacco retailers was obtained from the California Department of Tax and Fee Administration. The list contains detailed information on retailers who are currently licensed to sell tobacco and/or vaping products within the State of California and was collected in May 2019. Information from this list includes: (a) license number; (b) owner name; (c) doing business as (“DBA”) name; (d) retailer address; (e) date of license commencement; and (f) date of license expiry. The licenses obtained from CDTFA were cross-referenced using Yelp in order to identify tobacco store categories. Custom programming scripts were written in the computer programming language Python and were used to scrape publicly available store categories linked to Yelp business listings matched for CDTFA store names and addresses.

Businesses on Yelp are automatically assigned different business categories based on input from Yelp users or based on categorization from the platform’s data curation teams. Based on existing Yelp categories, the web scraper was used to classify tobacco retailers into the following categories: (i) specialized stores (categorized on Yelp as “Tobacco Shops” and/or “Vape Shops”) and (ii) non-specialized stores (i.e., convenience/grocery stores etc., that are licensed to sell tobacco and/or vape products). Specialized stores were further categorized as (i) tobacco and vape stores (categorized on Yelp as “Tobacco Shops” and “Vape Shops”); (ii) vape-specific stores (categorized on Yelp as “Vape Shops” only); and (iii) tobacco-specific stores (categorized on Yelp as “Tobacco Shops” only). Using the Microsoft Bing API (Application Programming Interface), the latitude and longitude for each of the retailer addresses was obtained for purposes of geolocation.

In order to test possible associations between retailer count and store category, smoking prevalence data (i.e., which includes prevalence for smoking and vaping nicotine) for the year 2019 were obtained from the 500 Cities project available from the CDC, a dataset that provides estimates for chronic disease risk factors, health outcomes, and clinical preventive services [20]. Additionally, data specific to vaping (nicotine) prevalence was obtained from the Esri Market Potential dataset using Esri’s Community Analyst tool [21]. Esri’s Market Potential data provides details about the products and services used by consumers and the database is based on survey data which provides the expected number of consumers and a Market Potential Index (MPI). The vaping prevalence data was obtained at the zip code level for California. Also, retailer count at zip code level had better gradient than at the census tract level. Hence, the smoking prevalence data obtained from CDC at the census tract level was aggregated to the zip code level for further analysis. Data for California state population was obtained from the American Community Survey at the zip code level [22] and was multiplied by smoking prevalence and vaping prevalence data to obtain smoking population and vaping population respectively at the zip code level.

### Data Analysis

A total of 22,131 retailer licenses were obtained from the CDTFA, out of which 15,270 (69%) were automatically categorized by cross-referencing with Yelp store names and addresses. The remaining retailer licenses were manually categorized by using the Yelp search function. ArcGIS v10.7.1 (Esri: Redlands, CA) was used to plot the point coordinates (latitude and longitude) of each retailer store on a California base map. These point coordinates were then aggregated to obtain the total number of retailers within each zip code, for each store category. Aggregated estimates of retailer count by zip code were exported to conduct further statistical analysis as better gradient and variation in distribution was obtained on analyzing the zip code retailer count as a continuous variable.

A series of simple linear regression tests were performed to test the association between tobacco and/or vape store retailer count and smoking population. The following categories of retailer count were used as the dependent variable in regression analysis: (a) specialized stores (number of stores specializing in the sale of tobacco products, vape products, or both); (b) tobacco-specific stores (number of stores specializing in the sale of tobacco products); (c) vape-specific stores (number of stores specializing in the sale of vape products and other emerging and alternative products); (d) tobacco and vape stores (number of stores specializing in the sale of both tobacco and vape products); and (e) non-specialized stores (number of non-specialized retail stores [e.g., convenience stores, grocery stores] that are licensed to sell tobacco and/or vape products). Regression analyses with these dependent variables were then replicated for the vaping population. Population normalized tobacco retail density was used for further geospatial analysis. Spatial clusters of high values (hot spots) and low values (cold spots) for all tobacco and/or vape retailer storefronts were mapped before and after normalization using optimized hot spot analysis tool in ArcGIS v10.7.1 (Esri: Redlands, CA). This tool calculates the Getis-Ord Gi* statistic and allows for the mapping of related z-scores. Spatial autocorrelation was assessed using Anselin Local Moran’s I tool which classifies the retail density areas into hotspots (high–high cluster), cold spots (low–low cluster), spatial outliers (high–low or low–high cluster), and non-significant areas. Visualizations using Anselin Local Moran’s I tool convey autocorrelation, thereby reflecting how values for a zip code are similar to adjacent zip codes, whereas visualizations using Getis Ord Gi* tool convey spatial clustering, thereby reflecting aggregations of high or low values. All statistical analyses were conducted using SPSS version 27. A *p*-value less than 0.05 was considered statistically significant.

## Results

**Fig. 1:**
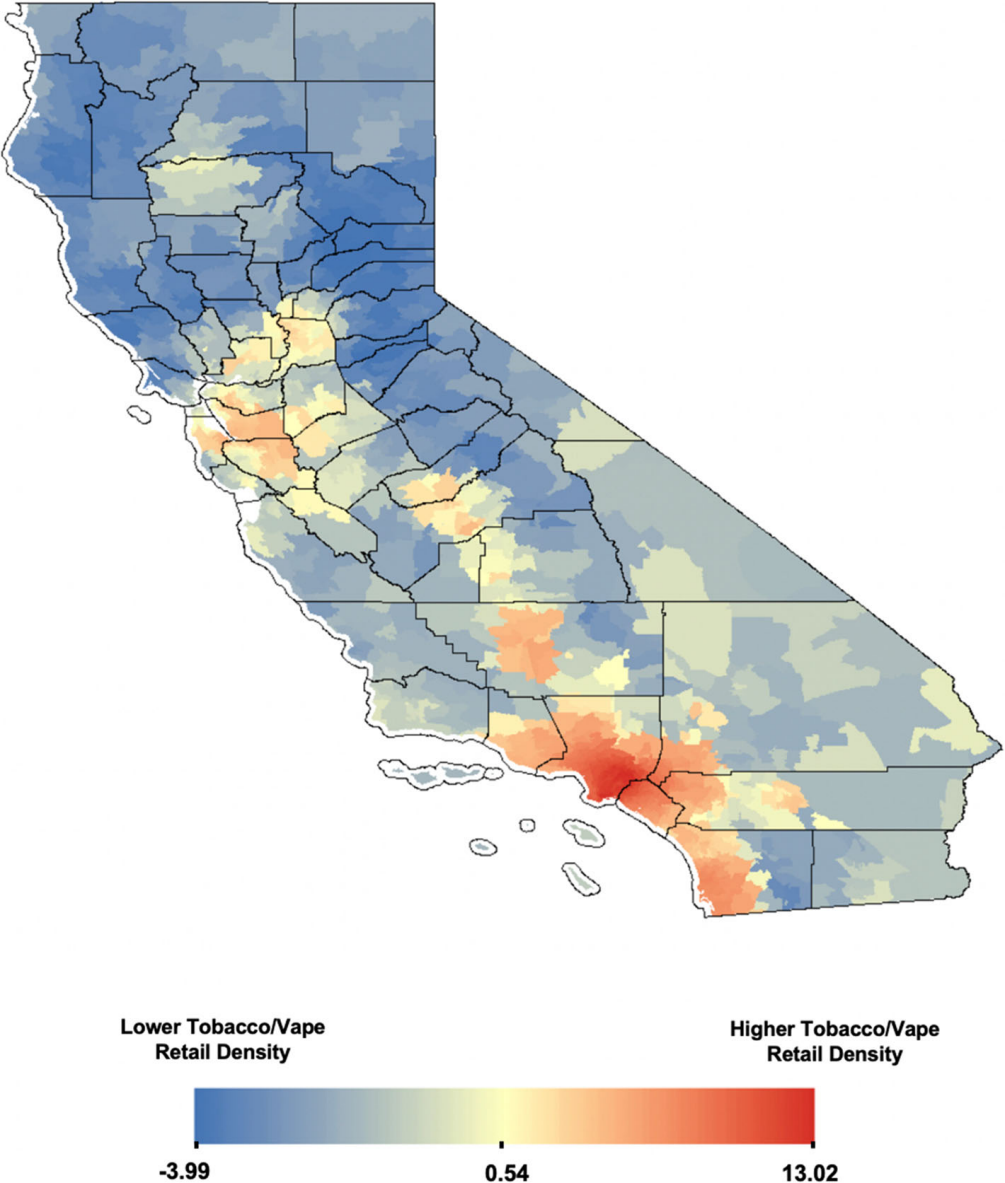
Non-normalized Z-scores for spatial hotpots of tobacco retailer count in California (2019)

**Fig. 2:**
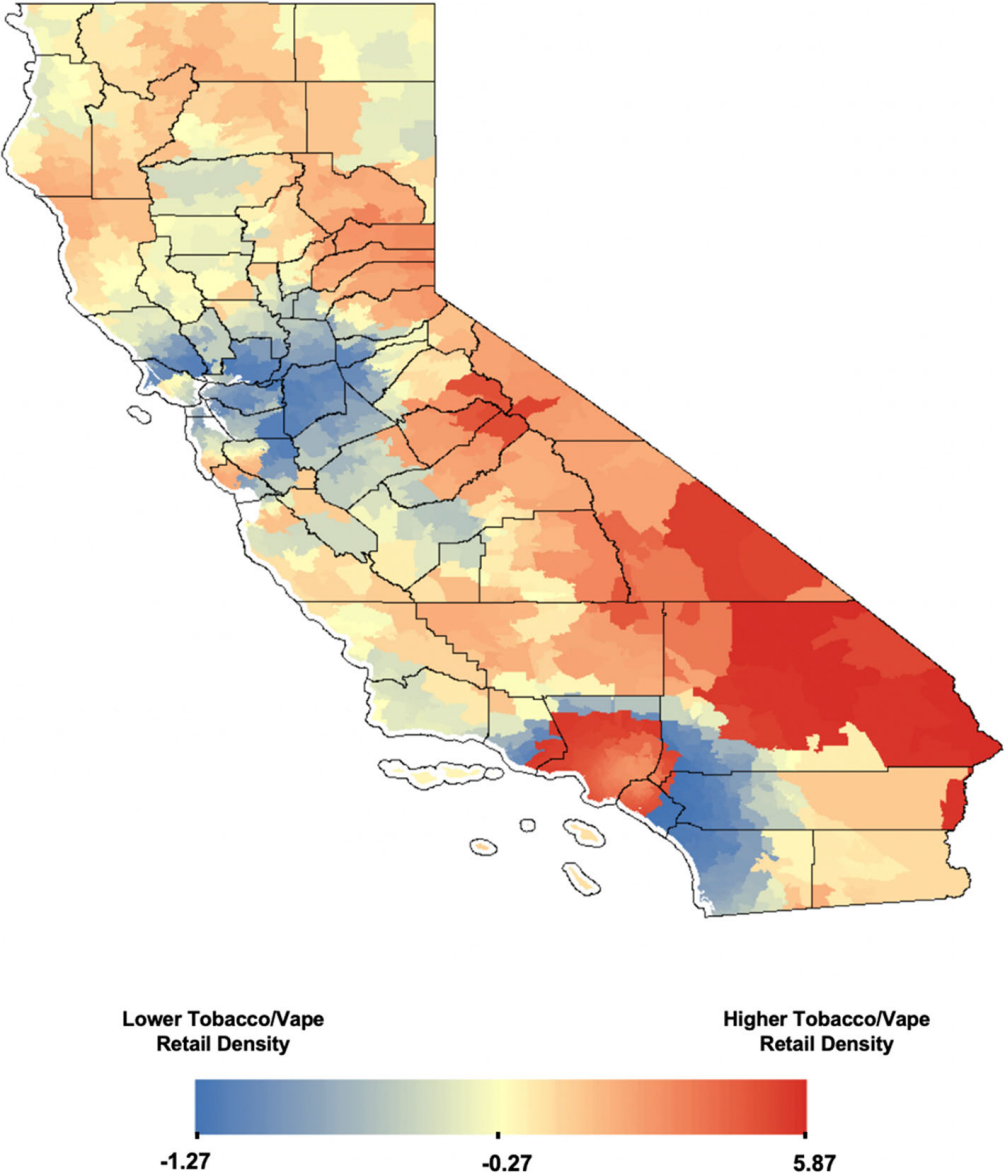
Population normalized Z-scores for spatial hotpots of tobacco retail density in California (2019)

**Fig. 3:**
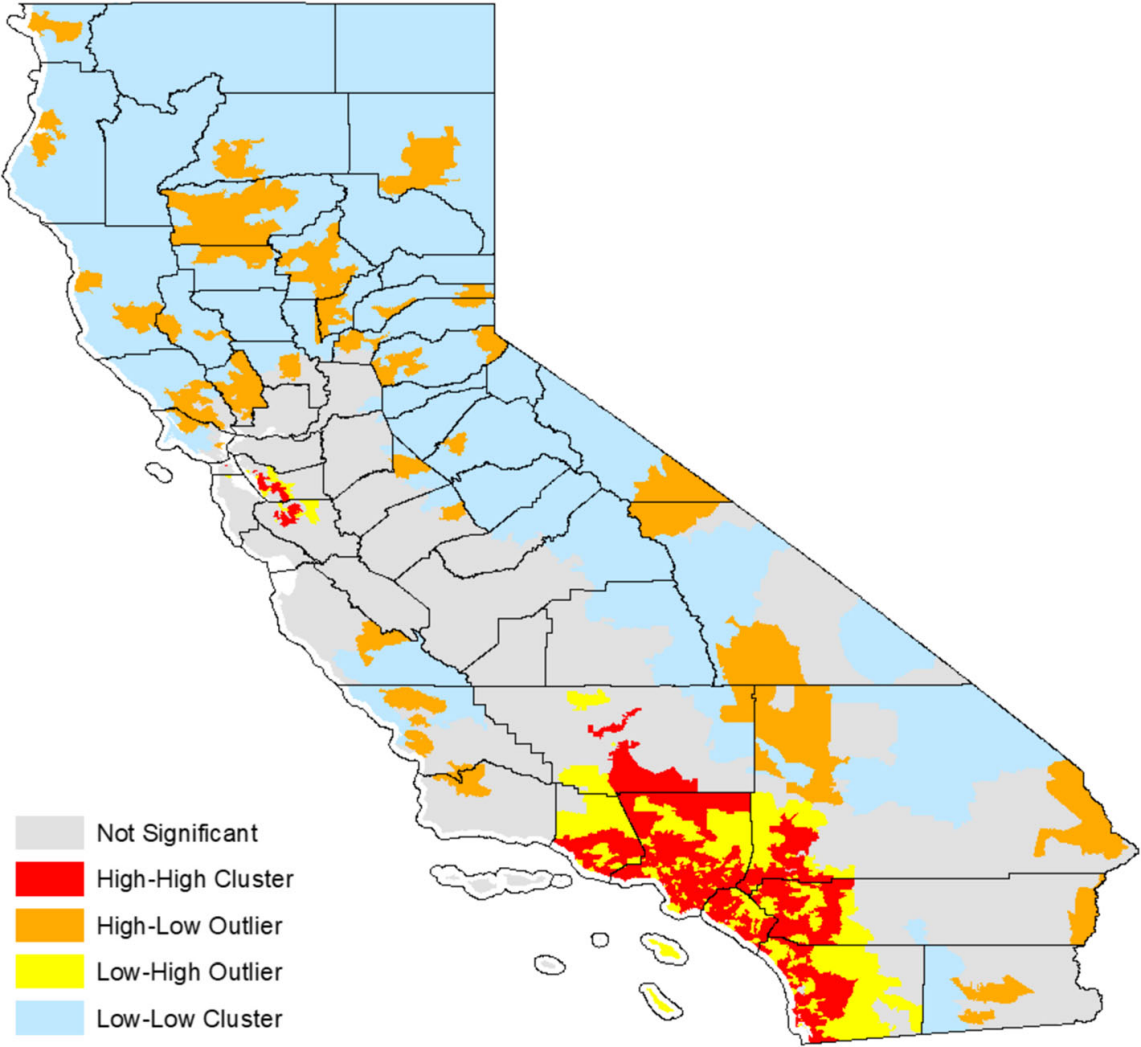
Tobacco retail density clusters and outliers by county based on Anselin Local Moran’s I statistic in California (2019)

A total of 22,131 retail storefronts licensed to sell tobacco products were cross-referenced using Yelp. Of these, 90.57% (*n*=20,044) stores were non-specialized retailers, 3.71% (*n*=820) were tobacco-specific stores, 3.83% (*n*=848) were vape-specific stores, and 1.89% (*n*=419) were stores that specialized in selling both tobacco and vape products. Visualization of z-scores across California revealed clustering of high z-scores (hot spots) in densely populated counties such as Los Angeles County, Orange County, and San Diego County before normalization (see Fig. 1). Twenty-six zip codes had more than 100 licensed tobacco and/or vape storefronts. The highest retailer count (*n*=138) was observed for zip code 92101 in San Diego County with 124 non-specialized storefronts and 14 specialized tobacco and/or vape storefronts. The clustering of high z-scores (hot spots) after population normalization was observed in Los Angeles County, but not in San Diego County (see Fig. 2). The Anselin Local Moran’s I showed statistically significant hotspot clustering of tobacco retail storefronts in 2019 in Los Angeles County, Orange County, and San Diego County (see Fig. 3).

Other zip codes with over 100 licensed tobacco and/or vape storefronts were located in San Diego County (92109, 92110), Orange County (92683, 92801, 92804, 92626, 92705), Santa Clara County (95110, 95112), San Bernardino County (91730, 91761, 91764, 92335), and Kern County (93304, 93307). No licensed tobacco and/or vape storefront was located in 794 zip codes. Three-hundred-ninety-four zip codes had between 1 and 25 stores, 291 zip codes had 26-50 stores, 198 zip codes had 51-75 stores and 66 zip codes had 76-100 stores.

All linear regression tests found statistically significant positive associations between smoking population and retailer count of tobacco and/or vape storefronts at the zip code level (*p* < 0.001) (see Table 1). The association between smoking population and retailer count of non-specialized storefronts licensed to sell tobacco and/or vape products had the highest effect estimate (β = 4.02). The proportion of variability in retailer count that was explained by smoking population was highest for stores in the non-specialized category (R^2^= 0.84) and lowest for storefronts specialized in selling both tobacco and vape products (R^2^= 0.35).

**Table 1:** Smoking population in association with tobacco retailer count by store type, California, 2019

Retailer count	Smoking Population Estimate (SE)	*p*-value	R^2^
Specialized stores	0.39 (0.009)	< 0.001	0.52
Tobacco and vape stores	0.09 (0.003)	< 0.001	0.35
Tobacco-specific stores	0.18 (0.005)	< 0.001	0.40
Vape-specific stores	0.19 (0.005)	< 0.001	0.45
Non-specialized stores	4.02 (0.04)	< 0.001	0.84

Similarly, all associations between vaping population and retailer count of specialized and non-specialized tobacco and/or vape storefronts at the zip code level were statistically significant (*p* < 0.001) (see Table 2). The association between vaping population and retailer count of non-specialized storefronts licensed to sell tobacco and/or vape products had the highest effect estimate (β = 12.24). The proportion of variability explained by vaping population was highest for the non-specialized store category (R^2^= 0.80) and lowest for storefronts specialized in selling both tobacco and vape products (R^2^= 0.35). While R^2^ values were comparable between models based on smoking population and those based on vaping population, vaping population explained a slightly lower proportion of variability in the distribution of non-specialized storefronts while generally explaining a slightly higher proportion of variability in the distribution of specialized storefronts.

**Table 2:** Vaping population in association with tobacco retailer count by store type, California, 2019

Retailer count	Vaping Population Estimate (SE)	*p*-value	R^2^
Specialized	1.30 (0.03)	< 0.001	0.59
Tobacco and vape stores	0.30 (0.01)	< 0.001	0.35
Tobacco-specific stores	0.60 (0.02)	< 0.001	0.47
Vape-specific stores	0.62 (0.02)	< 0.001	0.49
Non-specialized stores	12.24 (0.15)	< 0.001	0.80

## Discussion

This study focused on exploring the potential associations between smoking and vaping population and retailer count of specialized tobacco and/or vape stores in comparison with non-specialized storefronts that are licensed to sell tobacco products in the state of California. While the association was significant for all store categories, the proportion of variability in the retailer count of non-specialized storefronts explained by smoking/vaping population was substantially higher compared to that of specialized tobacco and/or vape shops. Modeling also suggested that specialized storefronts may have a slightly closer relationship with vaping than with smoking since vaping population explained a higher proportion of variability in the distribution of specialized storefronts.

Existing research provides evidence on positive associations between smoking and exposure to promotional advertisements at point-of-sale in tobacco retail environments for both specialized and non-specialized storefronts [23]. For example, according to a recent study on convenience store behaviors among youth and young adults, one-third of the participants purchased tobacco when visiting a gas station, suggesting that non-specialized retailers serve as critical access points in the acquisition and initiation of tobacco products [24]. However, variability in marketing strategies and advertisements in gas stations and other convenience stores may moderate these associations [25].

Prior research has also observed that prohibiting issuance of permits to any new tobacco retailer to operate within 1000 feet of a K–12 school or within 500 feet of another tobacco retailer reduced tobacco retail density in Santa Clara County [26]. Also, a case study in San Francisco observed that setting a cap on the maximum number of licenses can help reduce the disproportionate density of outlets in socioeconomically disadvantaged neighborhoods [27]. These prior studies point to the need for evidence-based tobacco control policies that can identify opportunities to reduce tobacco retail activity and in turn smoking prevalence [28]. Further supporting this conclusion, the association of tobacco retail density with smoking among adults has been observed regardless of exposure measure in a recent systematic review [29] and a significant positive association was observed between tobacco outlet density and smoking behavior among adolescents in a meta-analysis of studies examining tobacco outlet density around homes and schools [30].

Hence, these findings, along with results from this study, may suggest that more aggressive regulation using city zoning and licensing policies that restrict growth of tobacco retail outlets, particularly in areas where there is existing or trending high tobacco retail store count or density, may have a positive impact on reducing tobacco and vaping prevalence, though more research is needed that is small area and community specific. Identifying potential variations in the association between geographic retail density of specialized tobacco and/or vape shops and non-specialized tobacco vendors can also help optimize regulatory measures aimed at reducing vaping-related harms in communities with exceedingly high density relative to smoking and non-smoking population size.

### Limitations

This is an ecological study exploring the association between smoking/vaping population and tobacco retailer count for different store types and hence the results cannot be attributed to individuals. This study used the publicly available CDTFA listing of licensed tobacco retailers and did not include individuals (sole proprietors, husband and wife co-owners, and domestic partners) who are registered with, or hold licenses or permits issued by the CDTFA, per Civil Code Sect. 1798.69(a) of the Information Practices Act. The data was collected in May 2019 and included licensed tobacco retailers available as of this date. Hence, the study may have not captured the list of retailers who obtained a license during the year after the data collection, leading to possible incomplete licensure data for the year. However, the study included 22,131 licenses tobacco retail outlets, which is representative of the complete licensure data for the year and thereby reduced the likelihood of inadequate validity. Also, this study did not include any illegal tobacco and/or vape storefronts operating without a license from CDTFA and did not quantify the number of retail stores selling tobacco products without a license. Each retailer from the CDTFA listing was categorized based on Yelp data and ability for businesses to self-classify [31] which may lead to misclassification bias. Also, the store categorization based on Yelp was not cross validated through fieldwork or phone call verification. Further, the analyses were not adjusted for socioeconomic and other environmental factors which may be associated with tobacco retailer landscape and smoking behaviors. The data on vaping prevalence was obtained from Esri’s market potential data which is a survey-based database on consumer use of various products including e-cigarettes. The study did not ascertain the validation of this measure or the correlation between vaping prevalence and CDC’s smoking prevalence data. The study focused on testing the associations between tobacco retailer count and smoking/vaping population and should be considered hypothesis generating. Future research should focus on conducting neighborhood or community specific observational studies using resolute data differentiating vaping and smoking prevalence and individual tobacco retailer data including those selling without licenses taking into consideration sociodemographic factors influencing the tobacco retail landscape.

## Conclusions

Though exploratory, results from this study can help in formulating evidence-based tobacco control policies focused on scrutinizing and ultimately reducing tobacco retail activity on the basis of tobacco-related health harms in communities that have high retail density and tobacco/vape product availability and use. Findings can also extend to assessing the potential utility of more progressive retail restriction policies, including possibly extending permitting restrictions in high-risk areas (e.g., further prohibiting licenses/permits near schools, parks, colleges/universities, etc.) or ensuring that retail density and availability does not exceed a certain threshold. Tobacco regulatory science should also take into account if different licensure schemes are appropriate for different retail outlet categories, including experimenting with measures to reduce appeal and uptake generated by specialized stores and limiting convenience and access from non-specialized stores.

## Data Availability

The data that support the findings of this study are available upon request and certain data will be available freely from the website www.ghpolicy.org.

## Abbreviations

API: Application Programming Interface
CDC: Centers for Disease Control and Prevention
CDTFA: California Department of Tax and Fee Administration
DBA: Doing Business As; MPI: Market Potential Index
